# The borderline of bipolar: opinions of patients and lessons for clinicians on the diagnostic conflict

**DOI:** 10.1192/pb.bp.113.046284

**Published:** 2015-06

**Authors:** Emma Richardson, Derek K. Tracy

**Affiliations:** 1 Oxleas NHS Foundation Trust, London; 2 Institute of Psychiatry, King’s College London

## Abstract

**Aims and method** It has been observed that some individuals self-diagnose with a bipolar affective disorder and many are later diagnosed with a borderline personality disorder. There is a background context of clinical and neurobiological overlap between these conditions, and fundamental debates on the validity of current diagnostic systems. This qualitative study is the first work to explore the views of patients caught at this diagnostic interface. We predicted that media exposure, stigma and attribution of responsibility would be key factors affecting patient understanding and opinion.

**Results** Six core illness-differentiating themes emerged: public information, diagnosis delivery, illness causes, illness management, stigma, and relationship with others. Individuals did not ‘want’ to be diagnosed with a bipolar disorder, but wished for informed care.

**Clinical implications** Understanding patient perspectives will allow clinical staff to better appreciate the difficulties faced by those we seek to help, identify gaps in care provision, and should stimulate thought on our attitudes to care and how we facilitate provision of information, including information about diagnosis.

‘Someone call the doctor,

Got a case of a love bipolar’

– Katy Perry, ‘Hot N Cold’

Diagnosing mental illness can be clinically challenging. The publication of DSM-5^1^ and the surrounding debates have raised long-standing arguments about the fundamental validity of classification systems and their potential to variously stigmatise and disempower, or clarify and facilitate treatment and research.^2^ In parallel, neuroscientific research is increasingly moving towards the concept of ‘pathway’ illnesses^3^ with complex interactions between many risk genes and the environment, and fuzzier boundaries between conditions lying on a spectrum of phenotypes.^4,5^

Given the difficulties that trained professionals can experience, it is scarcely surprising that patients can have problems self-identifying psychiatric phenomenology and diagnostic labels, and indeed seeking expert help to establish a diagnosis has always been an important part of all healthcare. It has been noted recently that a growing number of individuals are presenting to mental health services having self-diagnosed with bipolar affective disorder (BPAD).^6^ In our experience, many are ultimately diagnosed with a borderline personality disorder (BPD).

Although there are obvious clinical similarities between the two conditions, notably mood cycling between pathological extremes, and a growing literature comparing them, to the best of our knowledge no one has previously tried to explore the opinions of those caught in this diagnostic dilemma. We hypothesised, fitting with the discussion piece by Chan & Sireling,^6^ that a complex combination of factors including stigma, causality and blame, celebrity culture, treatment (and treatability) and perception of staff attitudes would influence patient thinking regarding diagnosis. Furthermore, we anticipated that a better understanding of these issues would afford us greater insight into those we treat, with reflection for how this might positively affect our clinical practice in better communication, making and discussing diagnoses, and developing rational care plans.

## Method

### Participants

The study recruited eight individuals over a 3-month period in the London Borough of Bromley. All had self-diagnosed with BPAD, but were subsequently diagnosed with BPD. Four had no previous contact with mental health services and had been referred by their general practitioner (GP) to secondary mental health services for assessment due to their concerns that they had BPAD. Four had been within mental health services for varying periods of time (3-32 years): differing diagnoses had been suggested to them at different times, but a diagnosis of BPD had only been made for the first time in the month prior to interview and in the context of the patient having advocated for a diagnostic review in the belief they had BPAD. All participants were White women (7 British, 1 American), although this was not intended through study design, and were aged between 27 and 56 years old (median 35).

### Materials

A semi-structured interview was designed following a narrative review of the literature on the presentation and treatments of both BPAD and BPD. This was used to explore participants’ opinions on the similarities and differences between the two disorders in six areas: symptoms; the cause of the difficulties; public understanding; availability of clinical information; treatment; and stigma and attitudes. Participants were encouraged to express their thoughts on any topic they felt was important, including any not covered, or not fully covered, by the semi-structured interview.

### Procedure

The study received ethical approval through Oxleas NHS Foundation Trust. The London Borough of Bromley’s primary care liaison ‘intake’ team and the home and day treatment services were approached to identify eligible individuals. A letter of introduction outlining the study rationale was sent to 15 potential participants: 8 responded, were screened and deemed eligible, and provided informed consent to take part. All interviews were conducted jointly by both researchers and lasted between 36 and 75 minutes. They were transcribed verbatim (by E.R.) and processed

**Table 1:** The major themes for both diagnoses identified by participants

Theme	Bipolar affective disorder (BPAD)	Borderline personality disorder (BPD)
Public information on the illnesses	Highs and lows; euphoria; more predictable; public awareness; positive celebrity exposure; more internet resources and support groups	Quicker mood changes, more exhausting; highs ‘not really enjoyable’; unknown to the public; harder to diagnose

Delivery of the diagnosis	Given more time by staff; taken seriously	Mental health staff less knowledgeable; being kept in the dark; staff hesitancy; being dismissed; might present as clinically well; services geared towards the ‘most unwell’ rather than people with BPD

Illness causes	More genetic; brain ‘wiring’ or ‘chemical’ problem	More affected by the environment, especially early life traumas; inconsistent parenting

Illness management	Medications efficacious; psychology has less of a role; established protocols; a more passive process; staff better trained	Primary psychological management but treatments have limited effectiveness; never recover; needed more self-awareness and self-management; become one’s own therapist; a difficult process to effect personality change; symptoms mitigate against recovery; staff ‘anti-medication’ even where it worked

Stigma and blame	De-stigmatised by public exposure; received sympathy; outside one’s control; people might fear you	Reinforced by perceived staff attitudes and lack of information; staff hopelessness; personal fatalism; the name implies blame; responsible for being unwell; lack of sympathy; receiving a diagnosis could help remove some self-blame and provide better self-understanding

Relationships with others	Supported by friends, family and colleagues; can be concealed; infrequent nature would make it less troublesome	Insidious destruction and sabotage of relationships; the need for a good therapeutic relationship; sabotage professional care offered; seek out conflict; ever-present and cannot be concealed from relationships

through thematic analysis using the software package NVIVO v.10 for Windows.

## Results

Six main themes emerged: public information on the illnesses; delivery of the diagnosis; illness causes; illness management; stigma and blame; and relationships with others. Participants’ comments are summarised in Table 1.

### Public information on the illness

The largest theme to emerge was on ‘public information’: what these disorders ‘looked like’ and how one could find reliable information about them. All participants said they had more preceding knowledge of BPAD, primarily from mainstream media sources; most said they had never heard of BPD before being diagnosed and all thought the public at large would be quite ignorant of this diagnosis. With the hindsight of having been diagnosed with BPD, participants thought the two conditions were quite similar, with prominent problematic mood swings occurring more rapidly with a personality disorder. Several clinically delineating factors were suggested, fitting with the literature on the topic:^7–10^ ‘self-loathing’ was noted to be a core feature of BPD but not BPAD; a couple of participants opined that the rapidity of mood swings made BPD a more ‘exhausting’ illness, whereas four participants stated the ‘highs’ in BPD were not the pleasurable or euphoric type they imagined one might experience with BPAD:

‘With borderline personality it all happens within sort of minutes, rather than a few months and elated mood for a period of time and then the depression, within the space of an hour you can be down again... which is pretty exhausting.’

Most participants had made attempts to find information prior to and after accessing healthcare, most commonly though the internet. All had found useful information on BPAD, though with the caveat that this is what they initially supposed themselves to have, but only one participant said she found a useful online resource on BPD after being diagnosed. However, this finding, which was the subjective view of the small number of participants interviewed, can be challenged and there are certainly numerous professional and peer websites providing information, support and advice. Putatively, the sense of there being ‘less information’ might be a better reflection of the celebrity culture that was cited by seven participants as informing their views specifically of BPAD, and no participants could think of parallel examples of hearing about BPD:

‘[The public] haven’t a clue, never heard of [BPD]. If you went out now and took a questionnaire, a very simple yes/no questionnaire: ”Have you heard of?”, I bet you would get 90% ”no” as a response.’

### Delivery of the diagnosis

An interesting finding to emerge was that not one of our sample ‘wanted’ to ‘be bipolar’, with all regarding this as a serious illness with no degree of ‘social desirability’. The label of bipolar disorder had been self-affixed as a means of trying to understand the difficulties they faced, with a sense that it seemed a ‘best fit’. Three of those already in mental health services said they had an awareness that (at least some) staff disagreed with their self-diagnosis, but that they were not offered any alternative. Most said that when the BPD diagnosis was first put to them they did not have enough time to ask questions about this or talk through what it meant, and several used the word ‘abandoned’ in this context. Several participants initially challenged the diagnosis of BPD, but only in the context of feeling they were being dismissed or pejoratively judged by staff. One recalled a staff member saying ‘there’s nothing we can do for you’, whereas another said she was told, post-diagnosis ‘oh well, yeah, that’s a personality disorder, so we can’t really help that’. Several thought this was because staff ‘don’t know as much’ about BPD as they do about BPAD, and might ‘cover their ignorance’ through dismissive attitudes. Six participants acknowledged it might be more difficult for professionals to reach the diagnosis of BPD; that BPAD might ‘look more obvious’; and that the labile nature of BPD meant their presentations might be erratic – including individuals presenting as clinically well – which might confuse staff. One participant had been seen intermittently for varying durations by mental health services over a period of 30 years before a diagnosis of BPD was put to her. The discussion arose when she inadvertently saw the phrase on her psychiatrist’s computer screen:

‘It also makes me angry, not because I have got [BPD], but angry because I have been seen by mental health professionals over the years and no bugger has mentioned anything about this.’

All eight participants stated that when time and care were taken to explain what a personality disorder was, why it might occur, how it might manifest for different people, and how one might try to manage ensuing difficulties that the diagnosis of BPD ‘made sense’. In fact, the majority of patients described a sense of relief at having had a long-term difficulty named and contextualised, allowing them to think of how they might prospectively deal with it. Two participants said that they felt sufficiently strongly that the appropriate discussion of diagnosis with patients was so critical a professional training need that they were happy to volunteer time to speak to staff groups about this:

‘I felt absolutely over the moon because I had a real thing with a real name and I wasn’t being told I was just hysterical and imagining it... so yes, to find out is a huge relief, and it is not that I am a complete bloody arsehole... it wasn’t me being obnoxious or out of control as a person.’

There was unanimity in feeling that anyone diagnosed with BPAD would be given more time by staff to talk through the illness implications for them and their family, and that in such discussions professionals would be far less reticent and ‘take it seriously’. However, not all help-seeking interactions with staff were reported in negative terms: one participant recalled a very supportive one-to-one session with her key worker, shortly after she had received her diagnosis:

‘[He said] ”it’s something like having blue eyes, it’s nothing you can help and it’s nothing to be ashamed of, it is just the way you are and it’s treatable”, and he was very nice about it you know... it made me feel better’.

### Illness causes

The literature supports an important role for environmental factors, particularly early life trauma, in both BPD^11^ and BPAD,^12^ although sexual abuse rates may be greater in those with BPD.^13,14^ Twin studies have shown a high degree of heritability for BPD,^15^ although this is still less than that of BPAD.^16^ Fitting with this there was reasonable unanimity among participants that BPAD was ‘more nature’ and BPD ‘more nurture’, with BPAD seen as variously a brain, neurological or chemical disorder that one was more likely to inherit and BPD a condition that developed in light of environmental stressors and traumas, with particular emphasis given by most (5) participants to the notion of inconsistent or unloving parenting:

‘I have always thought that bipolar [disorder] was mainly a chemical imbalance of the brain and that to me it didn’t seem that it was... environmentally affected. Borderline [personality disorder] seems to me as less of a chemical problem and more of a behaviour problem or reaction to environment and experiences.’

### Illness management

Participants’ comments on illness management were, in the main, in line with the principles encapsulated in national guidelines.^17,18^ Most considered that medication was the cornerstone of treatment for bipolar affective disorders. In this way treatment for those with a bipolar illness was seen as a more passive process, wherein one could ‘just take the medication and get on with it’:

‘The way I look at it is, if someone is diagnosed with bipolar [disorder] and... you get to a stage where you work out what medication suits them, I am therefore assuming they would operate as a normal functioning human being. Now there isn’t a pharmacological proposition for the likes of us, then we have to carry on in our own world and have to just get on with it, so we can’t reach that level of normality, can we?’

Six participants expressed frustration that although they did not think medication was the primary treatment of BPD, staff had very negative views of issuing them any medication, certainly when compared with patients with BPAD:

‘I know you have this thing about why are people with [borderline personality disorder] given all this strong medication. From my experience I needed that to bring me down and keep my feet on the floor, because I was so impulsive and if I didn’t have that medication I probably wouldn’t be here as I would’ve jumped off a bridge or in front of a car.’

Participants thought that psychological engagement was more of a critical factor for BPD than BPAD, although interestingly five believed that the very nature of symptoms experienced in BPD mitigated against good outcomes: a labile mood could make it hard to predictably and consistently engage with therapy; and individuals might demonstrate impulsive sabotaging acts against those trying to help them that would ‘prove’ their worthlessness. One participant thought people with personality disorders could become ‘defensive and stubborn’ when offered advice, whereas another thought them ‘very sensitive’ to perceived criticisms compared with those with BPAD, and expressed her own general sense when speaking to staff that ‘I’ve tried everything and none of it works... it’s hard to imagine someone else can tell me how to deal with this’. Participants thought that individuals with BPAD were ‘more predictable’, whether having low or high mood, which would make it easier for the patient and clinician to engage and treat them.

### Stigma and blame

All participants thought that significant stigma surrounded all mental illnesses: individuals with both BPD and BPAD were seen as likely to experience prejudice, with, in broad terms, neither disorder clearly ‘better’ or ‘worse’, although there is a body of literature to suggest that BPD carries a particularly strong sense of stigma.^19^ The commentary by Chan & Sireling^6^ noted the potential role of public exposure, celebrity discussions and TV programmes in portraying BPAD in a positive light and our work reflected this nuanced aspect, with most participants saying such public discussions had helped de-mystify BPAD:

‘It is quite uplifting, you look at someone like Stephen Fry, because if he has got it and he is still getting out and about and having a career, it’s not so bad for a person, I know... but borderline, I mean I don’t know.’

Although the point was not explicitly raised by any participant, and indeed denied by several, it remains possible that this ‘celebrity culture’ and media portrayal of BPAD might have imbued this condition with an implicit degree of social desirability and association with positive attributes such as artistic creativity, and therein account for the fewer negative comments accrued when compared with BPD. Staff attitudes were also seen to more negatively impinge on BPD, with the lack of discussion leading to a sense that ‘there’s something wrong with [borderline personality disorder]’. The very term borderline personality disorder was described by four participants as being demeaning, with one noting that it felt like a judgement on her life even though ‘there are aspects of my personality which are lovely, you know, I can be quite funny and humorous’. In 2003 the Treatment and Research Advancements National Association for Personality Disorders (TARA-APD) campaigned to change the name and designation of borderline personality disorder in DSM-5; more recently an internet survey of 646 individuals diagnosed with BPD noted that a considerable percentage thought this should be renamed in DSM-5, potentially to include the terms ‘emotion(al)’ and ‘(dys)regulation’.^20^

A final delineating aspect with regard to stigma was a sense of attribution of blame: seven participants felt that they, staff and the public at large would regard someone with BPAD as a ‘victim’ of a serious mental illness, whereas those with BPD were more likely to be perceived as ‘perpetrators’ or creators of their problems, enhancing feelings of guilt, shame and self-loathing. Nevertheless, several participants noted that confirmation of the diagnosis of BPD had alleviated some of this self-blame, with a sense that they had ‘a real problem, like other people had’.

### Relationships with others

In discussion of the relationships with friends and family as well as professional staff, all participants felt this was a more difficult issue for those with BPD than for those with BPAD. Interestingly, in both cases participants felt blame could at least in part be attributed to those with a BPD, as well as to prejudicial attitudes:

‘they would be more understanding [of BPAD]... with borderline it is just these personality traits that are very difficult to live with... it is just a lot of work and you have to understand and I don’t think people can be bothered to try and understand other people.’

‘I seem to have this dependence on the therapist or psychiatrist... sometimes I would get really angry and lose my temper with people who are caring for me, I understand why professionals would dread [individuals with BPD] more than [those with] bipolar [disorder].’

In general, BPADs were seen as something that might be more easily concealed from others, whereas a personality disorder was too pervasive for this:

‘Nobody at her work knew [my friend] had bipolar. It has never been discussed, never been an issue, why? Because there has not been any abnormality of behaviour. But [people who have a borderline personality disorder] are doing it all the time.’

## Discussion

Both BPD and BPAD are common mental health conditions, affecting 4–12% (BPD)^21^ and 1–4% (BPAD)^22^ of the population, and of course they can occur comorbidly.^23^ For professionals there are apparent similarities between them, and several recent systematic reviews have explored this topic.^13,24,25^ As well as an overlap in symptomatology there are interesting data indicating that both conditions demonstrate some similar neurobiological changes, especially to the limbic system and in frontolimbic connectivity – although with differences in amygdalar and hippocampal alteration – and to serotonergic and dopaminergic neurotransmitter systems. Nevertheless, most work supports the concept that these two disorders are fundamentally distinct conditions.^7,13,26–28^ Despite this broad literature, to the best of our knowledge no previous work has explored the opinions of those caught in the diagnostic dilemma on the similarities and differences between the two disorders.

### Study limitations

Our study included only eight participants, all women and from a single London borough, and this may hinder the generalisability of our data. Furthermore, there might be a responder bias, and the opinions of the seven potentially eligible participants who declined to consider taking part might have been quite different. No clear differences in response were noted between those newly referred to mental health services and those already receiving care for some time, and the latter did not ‘know more’ about BPD. None of our participants were continuing to question their diagnosis of BPD, and all had had some time to contemplate it before the interview. There were more negative comments expressed about BPD, even if participants said they did not think this was a ‘worse’ condition. We did not identify, and are not aware of, any patients presenting with concerns that they have BPD only to be diagnosed with BPAD: this may be less likely due to the identified issue of public awareness. No viewpoints of those with BPAD on the difference between the disorders were obtained.

### Implications

Our study suggests that people do not ‘want’ to be diagnosed with bipolar affective disorders; they are looking for information and clear communication with professionals. Whereas previous work has qualitatively explored the thoughts and feelings of those diagnosed with BPD (and BPAD), none has evaluated a diagnostic interface and prior knowledge of the disorder. One cannot receive appropriate treatment for something one is unaware of, and there must be many individuals suffering psychological distress and the symptoms of BPD without being aware of the nature of their illness and struggling to define their difficulties.

At the broadest level, there are interesting questions about the role of the mental health professions and professional bodies such as the Royal Colleges of Psychiatry and Nursing and the British Psychological Society in the UK, third-sector organisations and the media in the discussion of mental health disorders. Such organisations, and many others, continue to roll out worthy campaigns to target stigma and discrimination in mental health. Information on specific mental health difficulties, including BPD, is available, including a leaflet produced by the Royal College of Psychiatrists and designed to be read by non-professionals (http://www.rcpsych.ac.uk/healthadvice/problemsdisorders/personalitydisorder.aspx). However, a critical question is how could one look for what one does not know exists? Most participants noted that their information about mental ill health came, at least initially, from general media and in particular from awareness of celebrities whose mental health difficulties had been well documented. Stephen Fry was held out as a particularly positive role model by most of our participants: his willingness to talk publicly and openly was cited as being both inspiring and informative, and had a marked impact on participants’ conceptualisations about their own problems, including influencing their thoughts on their diagnoses. A perhaps unanswerable question is how to achieve a similarly positive and educational context for BPD. Whether campaigns such as that by TARA-APD have significantly raised the profile of BPD remains uncertain.

Nevertheless, the challenge to mental health staff faced with patients in this diagnostic dilemma is clear. Our patients are asking us for information, for time to think about and question what we say, and for the respect of being treated honestly in such discussions. A recent review by Gask *et al*^9^ noted the critical importance of hope, optimism and an accessible ‘trusting relationship with an open, non-judgemental manner’ when managing personality disorders.

Diagnosis is part of healthcare, and while important debates about the validity of our existing models continue – and the British Psychological Society expressed concern in 2011 about the potential medicalisation of what might be considered normal variation in behaviour^29^ – a diagnosis can help conceptualise difficulties and instigate appropriate and evidence-based care. Although professionals can be circumspect about making a diagnosis too rapidly (and many psychiatrists have been traditionally taught not to diagnose a personality disorder on first assessment), there is a very real danger that failure to do so can hinder care and mean that individuals receive no, or inappropriate, treatments that might not help, and indeed that might cause harm. If we are withholding or being unduly circumspect and hesitant about diagnosis, then we must ask ourselves why, and consider how our (in)actions might make a patient feel. Failure to openly discuss diagnostic thoughts risks perpetuating stigma and self-blame that can already be a common part of BPD. All our participants stated that having an accurate diagnosis was a hugely important step in self-reflection and understanding, and in considering their future, even if it came with other negative aspects.

In our sample several participants acknowledged that the diagnosis of a BPD might be hard to make; that the inherent lability could make it difficult to accurately assess the mental state and risk; and that the very nature of the symptoms suffered could make it challenging to consistently engage with a therapeutic programme and the staff providing care. There are real professional dangers of negative counter-transference in such situations and of projecting our frustrations or disappointments – current or historically accumulated – on those we treat, potentially furthering a sense of abandonment and rejection. Trust and the therapeutic alliance is a critical component of the relationship and process of our engagement with all patients, even if not addressed explicitly, and seldom more so that those with BPD,^30^ many of whom have had a significant history of past abandonments. We must be careful in suppositions that people are ‘choosing’ or ‘want’ diagnoses to ‘escape’ or deny a personality disorder: our data would not support such a hypothesis.

We believe there are many positives for patients and staff to take from this work. The debates on diagnostic systems and the neuroscientific research will continue, but what is being asked for is freely available: open and honest discussion, respect and information. Disagreements are part of clinical life and outcomes are not always as optimal as one would like: however, these factors can only be worsened by not listening. Our attitudes and self-reflection are vital: it is an interesting fact that BPAD is often conceptualised as a ‘serious mental illness’, but BPD is not, when the evidence suggests functioning and prognosis can be as bad in the latter.^24^ Few staff working in mental health can be unaware of the frequency and often profound severity of BPD, but there is a critical issue of perception: of allowing those we try to help to see our concerns, and making them feel listened to in clear dialogue. Borderline personality disorder was initially named as it was felt to ‘border’ on a psychotic state, but perhaps bordering on a bipolar one would be more apposite.
